# Environment-based preference selection promotes cooperation in spatial prisoner’s dilemma game

**DOI:** 10.1038/s41598-018-34116-0

**Published:** 2018-10-23

**Authors:** Yu’e Wu, Shuhua Zhang, Zhipeng Zhang

**Affiliations:** Coordinated Innovation Center for Computable Modeling in Management Science, Tianjin University of Finance and Economics, Tianjin, 300222 China

## Abstract

The impact of environment on individuals is particularly critical. In evolutionary games, adopting the strategy of the neighbor who performs better is nontrivial for the survival and maintenance of cooperation, in that such an action may help the agents to obtain higher benefit and more obvious evolutionary advantages. Inspired by this idea, we investigate the effect of the environment-based preference selection on the evolution of cooperation in spatial prisoner’s dilemma. A simple rule, incorporating individual preference selection via an adjustable parameter *α* to explore how the selection of the potential strategy sources influences individual behavior traits, is considered. Because social interaction may not be the only way of generating payoffs, we assume that the individual’s income is also affected by the environment. Besides, taking into account individual differences, we introduce the heterogeneity of the environment. Through numerous computing simulations, we find that environment-based preference selection, which accelerates the microscopic organization of cooperator clusters to resist the aggression of defectors, can truly promote cooperation within a large range of parameters. Our study indicates that the combination of heterogeneity and preference selection may be key for the sustainability of cooperation in structured populations.

## Introduction

Cooperative behaviors exist widely in biological and social systems, ranging from microorganism groups to complex human societies^1–5^. However, cooperation is costly: a cooperator has to pay a cost for benefiting others. These cooperative behaviors are inconsistent with Darwinism^6–8^: self-interested behaviors will be rewarded during fierce competition among individuals, but how can natural selection produce cooperation? Understanding the evolution of cooperation among selfish individuals in human and animal societies remains a grand challenge across a myriad of disciplines^9–12^. The puzzle of cooperation has plagued scientists, especially evolutionary biologists over several decades.

Evolutionary games are employed frequently as the theoretical framework of choice to interpret the appearance and maintenance of cooperative behavior^13–17^. As the essential yet minimalist example of a social dilemma, the prisoner’s dilemma has been widely employed for investigating the origin of cooperation^18–22^. In the original two-person one-shot game, two players simultaneously decide whether to cooperate (C) or to defect (D). Their payoffs depend on the simultaneous decision of both sides of the game. They both receive *R* upon mutual cooperation, which corresponds to the highest collective income (2*R*). Both the agents obtain *P* upon mutual defection. A defector receives *T* (the temptation to defect) when exploiting a cooperator, and the exploited cooperator gets *S* (the sucker’s payoff). For the prisoner’s dilemma game (PDG), the payoff ranking is *T* > *R* > *P* > *S*, which implies that the best strategy for each agent is to defect regardless of the opponent’s decision in one single round^23,24^. Although the collective will be better off if they both cooperate, defection is the evolutionarily stable strategy (ESS). The prisoner’s dilemma depicts the conflict of interest between the individual and the whole group. Therefore, specific mechanisms for interpreting the emergence and sustainability of cooperation are proposed, for example, different coevolution setup^25–28^, reputation^29–31^, reward^32,33^, punishment^34–37^, different evolution dynamics^38,39^, heterogeneity^40–45^, and so on. Nowak attributed all theses to the following mechanism: kin selection, direct reciprocity, indirect reciprocity, network reciprocity, and group selection^46^.

Traditionally, evolutionary games are investigated in an infinite, well-mixed population (complete graphs), where all players interact equally likely with each other^47^. As a matter of fact, fully-connected graphs constitute rather unrealistic representations of real-world network of contacts, in which one expects coexistence of local connections (spatial structure) and long-range connections, traits recently identified as characteristic of many natural, social, and technological network of contacts. Therefore, considerable attention has been shifted into spatial games and multitudinous works on the evolution of cooperation in structured populations spring up^48–51^. Especially, evolutionary graph theory provides a convenient framework for describing population structures: vertices denote players and edges indicate links between players in terms of game dynamical interaction^52^. The players populated on the vertices are constrained to play with their immediate neighbors along the edges. It is recognized that graph topologies play a significant role in the evolution of cooperation. As expected, the evolution of cooperation has been extensively explored in a variety of topologies such as regular square lattices, Erdös-Rényi (ER) graphs, small-world networks, Barabási-Albert scale-free (SF) networks and so on^53–58^. In particular, Nowak and May seminally introduced the spatial structure into the PDG, where the players were located on the square lattices and their payoffs were gathered from playing the game with their nearest neighbors^59^. The players were allowed to copy the strategy of one of their neighbors, provided its payoff was higher. It was demonstrated that the evolution of cooperation could be greatly promoted by spatial structure even without the aid of additional mechanisms or strategic complexity. The seminal works of Nowak and May have spawned many studies and new approaches in the evolutionary games on complex networks^60,61^.

At present, preference selection has received great attention and been proved to be an efficient way for favoring the evolution of cooperation^62–64^. Furthermore, in traditional evolutionary games, almost all the agents are usually considered homogeneous. However, individuals in reality are different, in other words, individuals are surrounded by neighbors with different personalities, suggesting that individuals are in a heterogeneous environment. Actually, the overwhelming evidence shows that heterogeneity, almost irrespective of its origin, promotes cooperative actions both theoretically and empirically^65^. For instance, Santos *et al*. indicate that scale-free networks with strong heterogeneity of degree distribution can provide a unifying framework for the appearance of cooperation^66,67^. On the other hand, because social interaction may not be the only way of generating payoffs, the impact of heterogeneous environment will be reflected in the individual’s income, which is reasonable and feasible. Thus, an interesting question appears: if we consider both heterogeneity and preference selection in one mechanism, how will cooperation evolve? In the present work, a mixed mechanism that includes preference selection and heterogeneity is introduced to the evolutionary PDG. In the model, individuals are heterogeneous, and thus each individual is in a heterogeneous environment. A parameter *u* is introduced to adjust the weight of earnings from the environment to the total fitness. The simulations are conducted in the PDG on the square lattice with periodic boundary conditions. As we show, appropriate preference selection parameters and larger heterogeneity proportions are more conducive to promoting cooperation.

## Results

In this paper, we introduce the preference selection and the individual heterogeneity in the spatial PDG. The introduced preference selection is related with the choice of imitating objects when the strategy is updated. The preference selection parameter is denoted by *α*. The heterogeneity is reflected in the heterogeneous environment in which the individuals are heterogeneous. We adopt the average of heterogeneity of the individual’s neighbors to represent the environment in which the individual situated. In reality, many different factors contribute to the overall fitness of an individual, and game gain are just one of those factors. Therefore, the parameter *u* is introduced to represent the proportion of the environment’s contribution to the individual’s fitness. Moreover, inspired by the previous work on system size effects^68^, we have conducted the model on larger size networks (200 × 200 and 400 × 400) and found that the simulation results are consistent with those on the network with the size of 100 × 100. For details of the model, see the method section.

**Figure 1:**
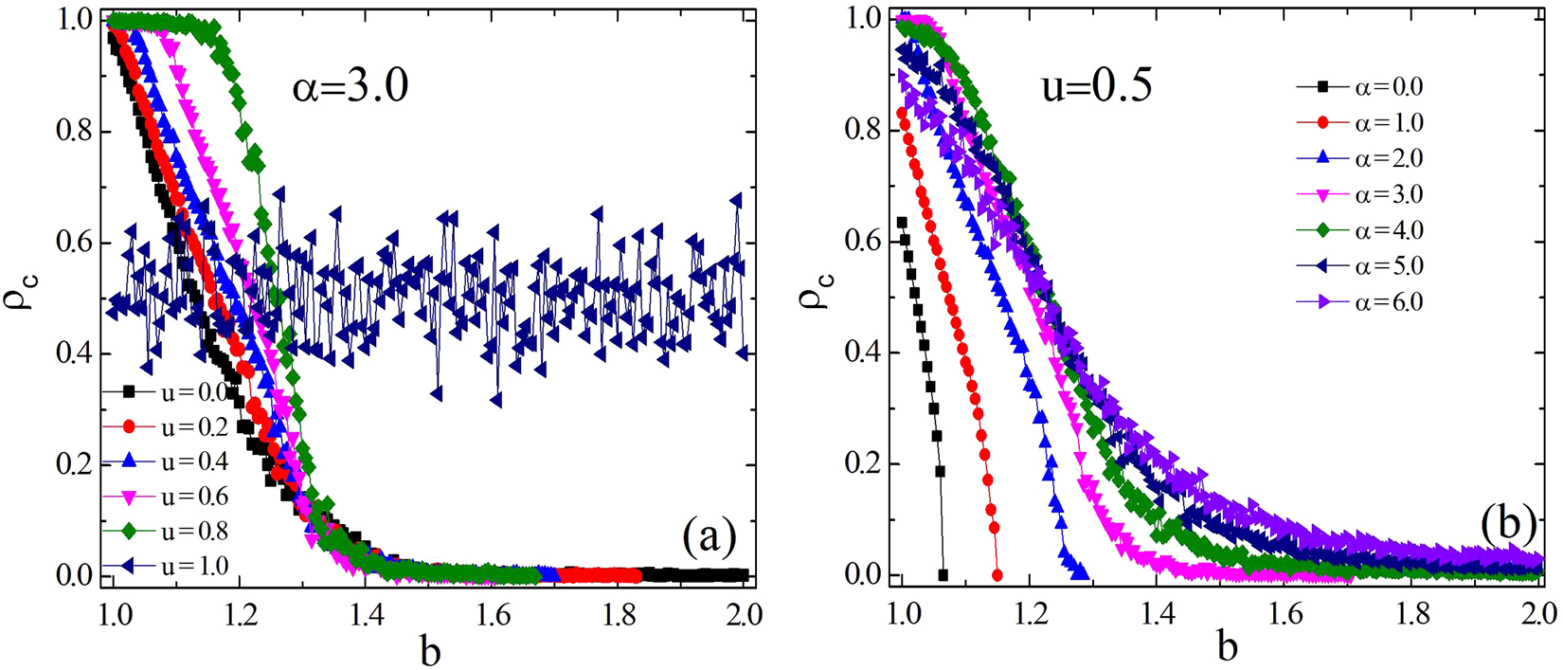
The fraction of cooperation *ρ*_*c*_ in dependence on the temptation to defect *b*. Panels (a) and (b) represent the conditions of changing the parameter *u* for *α* = 3.0 and regulating the parameter *α* for *u* = 0.5, respectively. All the results are obtained for *L* × *L* = 10^4^ nodes, *k* = 4 and *K* = 0.1.

It is instructive to first examine the influences of parameter *u* (*α*) on the evolution of cooperation. Figure 1(a) in which the parameter *α* is fixed at 3.0 presents how *ρ*_*c*_ varies in dependence on the temptation to defect *b* for different values of parameter *u*. When *u* = 0, the way that individual gains income through playing games will return to the traditional case. Under this parameter configuration, individual is only affected by the preference selection effect, and the evolution of cooperation has been improved to some extent. For *u* = 1, the individual’s fitness is entirely determined by the heterogeneous environment. The probability that an individual imitates the strategy of each of its neighbors is determined, and the evolution of cooperation is independent of *b*. Under this setting, the learning process will not be reflected in the entire evolutionary game and the fraction of cooperation versus the dilemma strength *b* fluctuates around 0.5, as shown in Fig. 1(a). When 0 < *u* < 1, the individual behavior is influenced by environmental heterogeneity and preference selection effects together, and its fitness includes incomes from the environment and benefits from game interaction. As presented in panel (a), the fraction of cooperation is proportional to the parameter *u*, which characterizes the percentage of benefits from heterogeneous environments to the fitness. This result confirms the truth that heterogeneity does promote the evolution of cooperation. Figure 1(b) depicts the fraction of cooperation *ρ*_*c*_ in dependence on the temptation to defect *b* for fixed *u* (*u* = 0.5) and changing *α*. When *α* = 0.0, individual will randomly select a neighbor to copy its strategy. For *α* > 0.0, the heterogeneous preference selection is introduced. The probability of an individual choosing a neighbor to imitate its strategy is positively related to the neighbor’s own characteristics *h*_*x*_ (see the Method section for details). For the small value of *b*, the proportion of cooperation increases first and then decreases with the value of *α*, indicating the existence of the optimal *α* value for promoting cooperation. While for larger *b* values, the proportion of cooperators is proportional to *α* within the selected range of parameters. We can draw the conclusion that when considering the preference selection mechanism, regardless of the value of *α*, the evolution of cooperation is promoted.

**Figure 2:**
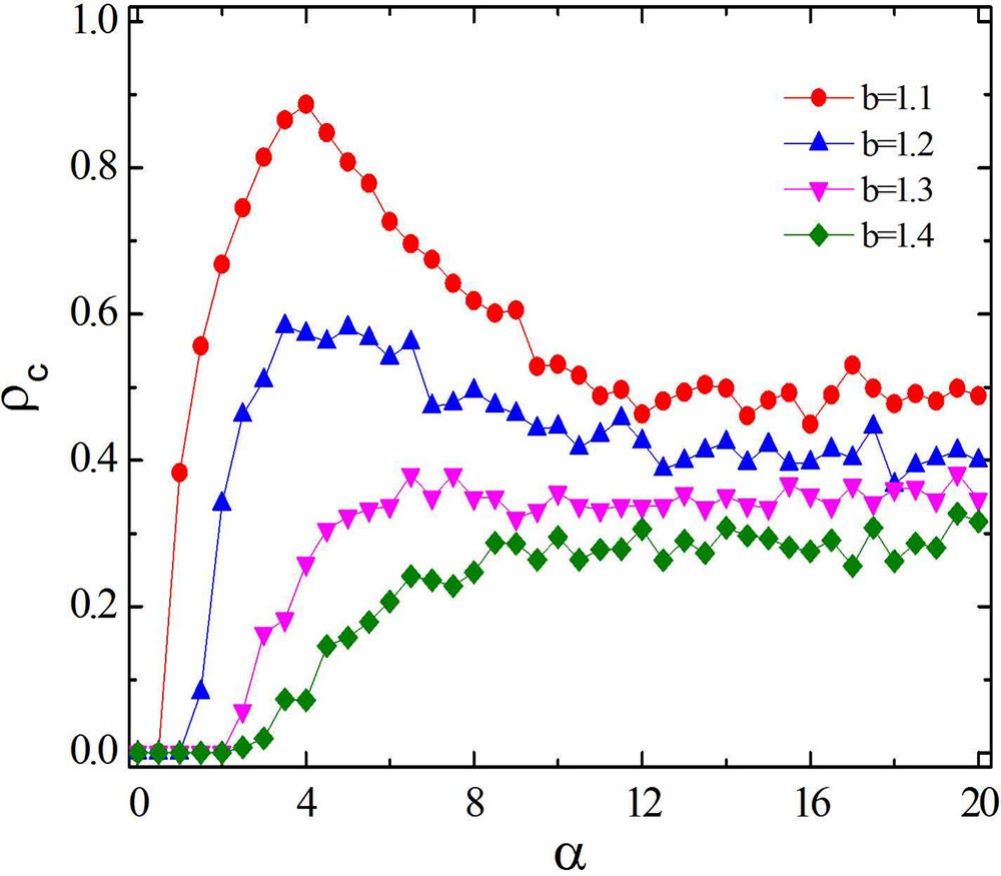
The fraction of cooperation *ρ*_*c*_ as a function of the preference selection parameter *α*. The parameter *u* is fixed at 0.5. Other parameters are consistent with those in Fig. 1. It is observed that for each determined *b*-value, when *α* > 12.0, the concentration of cooperation will be maintained at their respective values.

To explore the detailed impact of *α* on the evolution of cooperation, we present in Fig. 2 the density of cooperators *ρ*_*c*_ versus *α* for different *b* values. The parameter *u* is fixed to be 0.5. It is observed that the effect of parameter *α* on cooperative evolution is consistent with that expected in Fig. 1. For smaller *b* values, there seems to be a coherent resonance (i.e. a bell-shape curve): a specific *α* value generates the largest *ρ*_*c*_. Interestingly, the optimal *α* values are all between 2.0 and 6.0. For larger *b* values, when *α* is relatively small (*α* < 2.0), the fraction of cooperators is zero. While the concentration of cooperators *ρ*_*c*_ increases as the parameter *α* continues to grow. However, there is no bell curve for this parameter setting. Moreover, when *α* is larger than 8.0, the fraction of cooperators almost fluctuates around a certain value at each *b* value. The larger the value of *b* is, the more obvious this effect is. Typical examples are the results for *b* = 1.3 and *b* = 1.4, which are shown by the pink and green dotted lines in Fig. 2, respectively. In addition, it is worth noting that for each of the determined *b* values, the promotion effect of *α* on the evolution of cooperation is approximately constant when *α* > 12.0. Therefore, when we investigate the impact of the parameter *α* on the evolution of cooperation, it only needs to limit *α* to less than 12.0.

**Figure 3:**
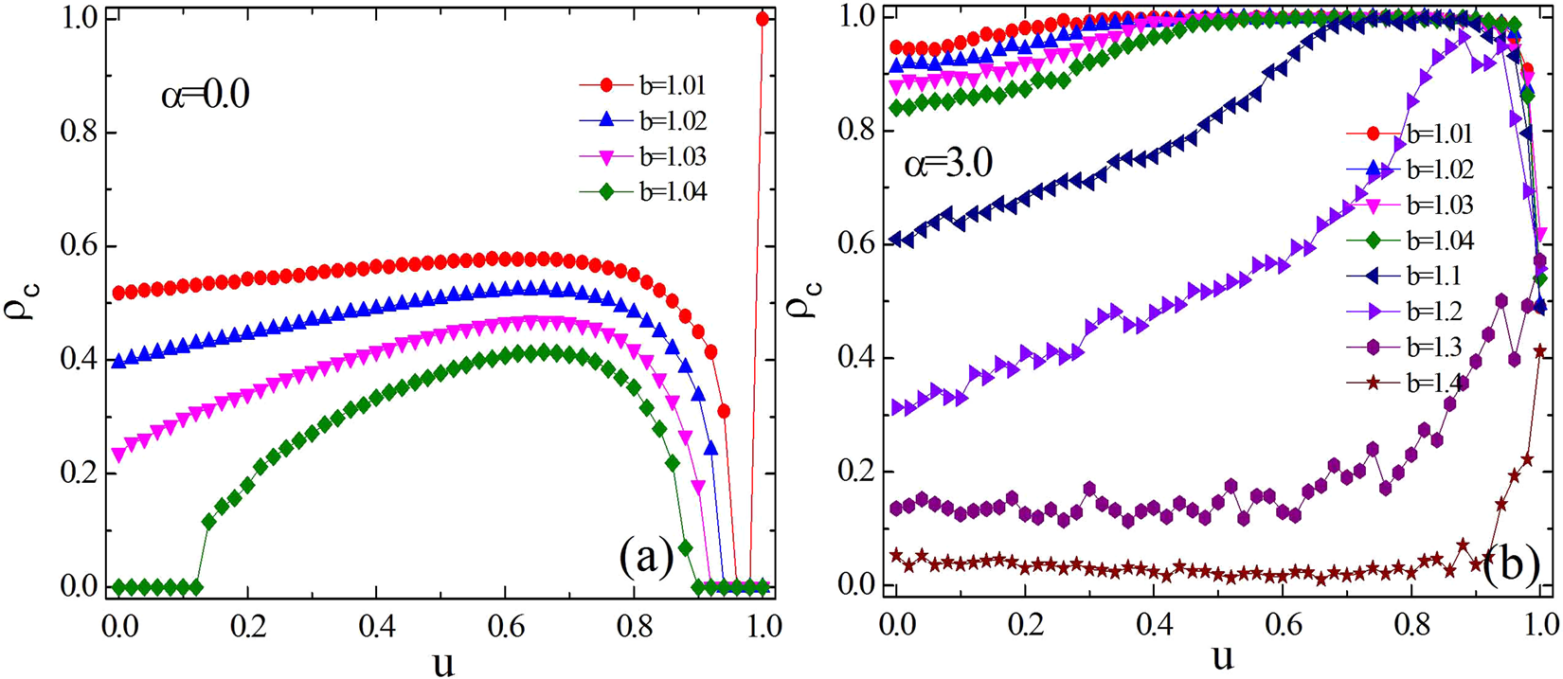
The fraction of cooperation *ρ*_*c*_ versus the parameter *u*. Panels (a) and (b) represent the results for *α* = 0.0 and *α* = 3.0, respectively. Both panels show that cooperation is best promoted for larger *u* values, irrespective of which *α* applies. Panel (b) suggests that the parameter *u* can enable cooperators to reach their exclusive dominance when *b* is relatively small.

Along this line, we next present the fraction of cooperators versus *u* for fixed *α* value as shown in Fig. 3. The results in panel (a) and panel (b) represent *α* = 0.0 and *α* = 3.0, respectively. When *α* = 0.0, the focal player will randomly pick up one neighbor to copy its strategy. It is observed from the figure that the fraction of cooperators rises with the increase of parameter *u* and reaches the maximum value around *u* = 0.7. Compared to the traditional version, the concentration of cooperators in equilibrium has been heightened to some extent, suggesting that the introduction of heterogeneity of the agent does promote the evolution of cooperation even if the imitative neighbor is randomly selected. When *α* equals to 3.0, both the preference selection and the individual heterogeneity are considered. As presented in Fig. 3(b), we can intuitively observe that the evolution of cooperation has been significantly improved under this combination mechanism. For instance, when *b* = 1.1, cooperators dominate at smaller *u* values and even displace defectors for larger *u* values. However, in panel (a), for the same *b* value, the largest fraction of cooperators is around 0.6, which demonstrates that the combination mechanism is more conducive to cooperation, and the fraction of cooperators is proportional to the parameter *u* for this parameter configuration.

**Figure 4:**
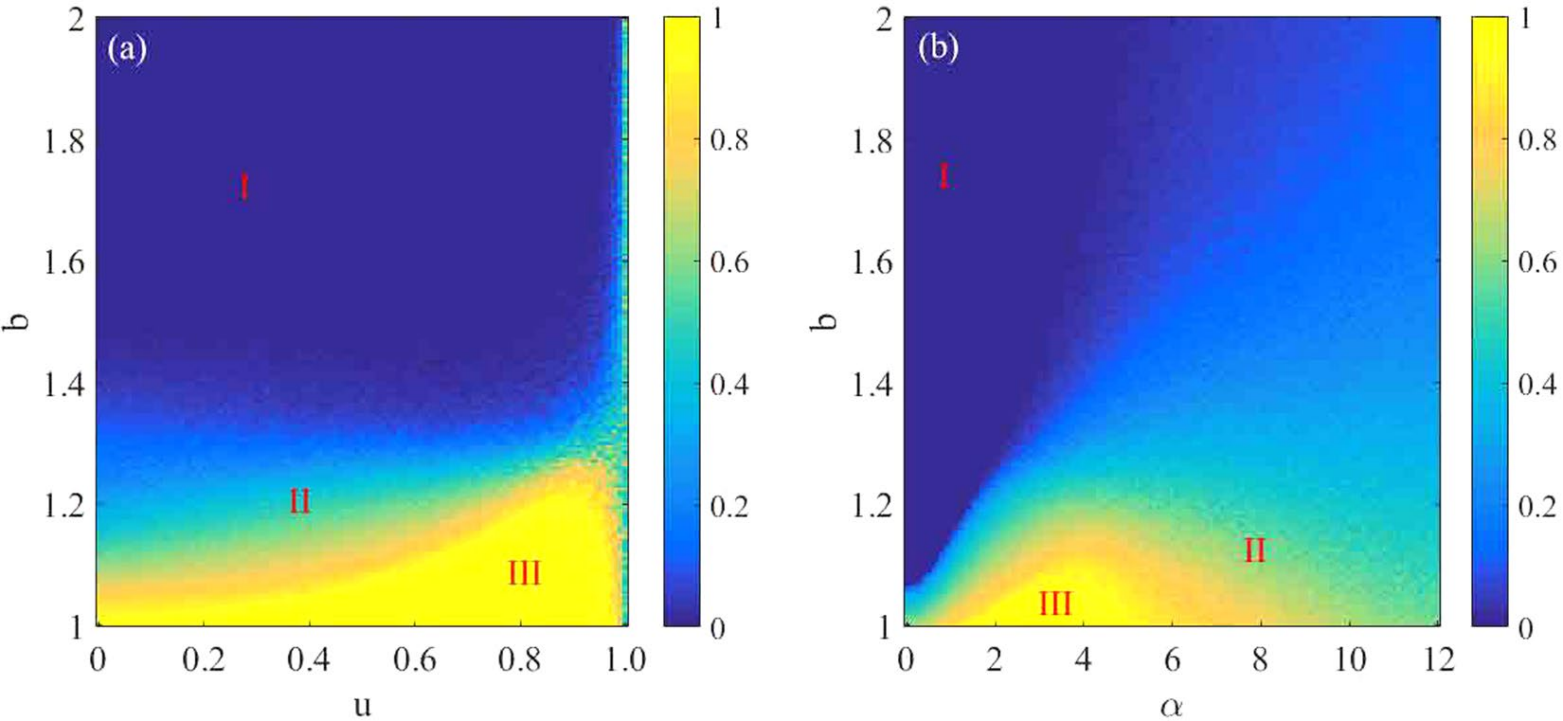
The color-coded (see bar on the right) fraction of cooperation *ρ*_*c*_ on the *b*-*u* parameter plane and the *b*-*α* parameter plane. Panel (a) represents the *b*-*u* parameter plane for *α* = 3.0 and panel (b) shows the *b*-*α* parameter plane at *u* = 0.5. The simulation results suggest that in addition to *u* = 1.0, the fraction of cooperation is positively correlated with the value of *u* when the value of *α* is fixed. Panel (b) shows that when *α* is between 2.0 and 6.0, cooperation has been improved to the most obvious.

Actually, it is particularly important to investigate the properties of phase transition when exploring the evolution of cooperation^69^. To fully and comprehensively explore the influences of the parameters *α* and *u* on the evolution of cooperation, we present in Fig. 4 the color map encoding the fraction of cooperation *ρ*_*c*_ on the *b*-*u* parameter plane in panel (a) and the *b*-*α* parameter plane in panel (b). In the *b*-*u* parameter plane, the parameter *α* is fixed at 3.0. As shown in Fig. 4(a), the whole plane is divided into three phases: full defection phase (phase I), well-mixed phase for cooperation and defection (phase II) and full cooperation phase (phase III). When *u* = 0.0, the agent’s fitness merely includes game gains, and the preference selection mechanism is considered only, which somewhat favors the evolution of cooperation. As discussed above, when *u* = 1.0, the density of cooperators which is displayed by the green bar in Fig. 4(a) fluctuates around 0.5. When 0 < *u* < 1, the survival of cooperators will become relatively easy. For small and middle *u* values, cooperators dominate in the *b* range from 1.0 to about 1.1. For larger values of *u*, the frequency of cooperation can still be maintained at more than 80% even when *b* reaches around 1.3. Increasing *u* enhances the role of environmental heterogeneity, which plays a crucial role in promoting cooperation. In the *b*-*α* parameter plane, the parameter *u* is set to be 0.5. When *α* = 0.0, corresponding to the case where the simulated object is randomly selected, as presented in panel (b), cooperators die out soon even when the heterogeneity of the environment is introduced. When *α* > 0.0, that is, both the effects of heterogeneity of the environment and preference selection are taken into account, the enhancement of cooperation is obvious, especially when *α* is between 2.0 and 6.0. In particular, cooperators even can survive through the entire range of *b* for some *α* values. Based on the above observations, we can conclude that the combination of heterogeneity of the environment and individual preference selection greatly promotes the evolution of cooperation, although each of them can also favor the evolution of cooperation to some extent.

**Figure 5:**
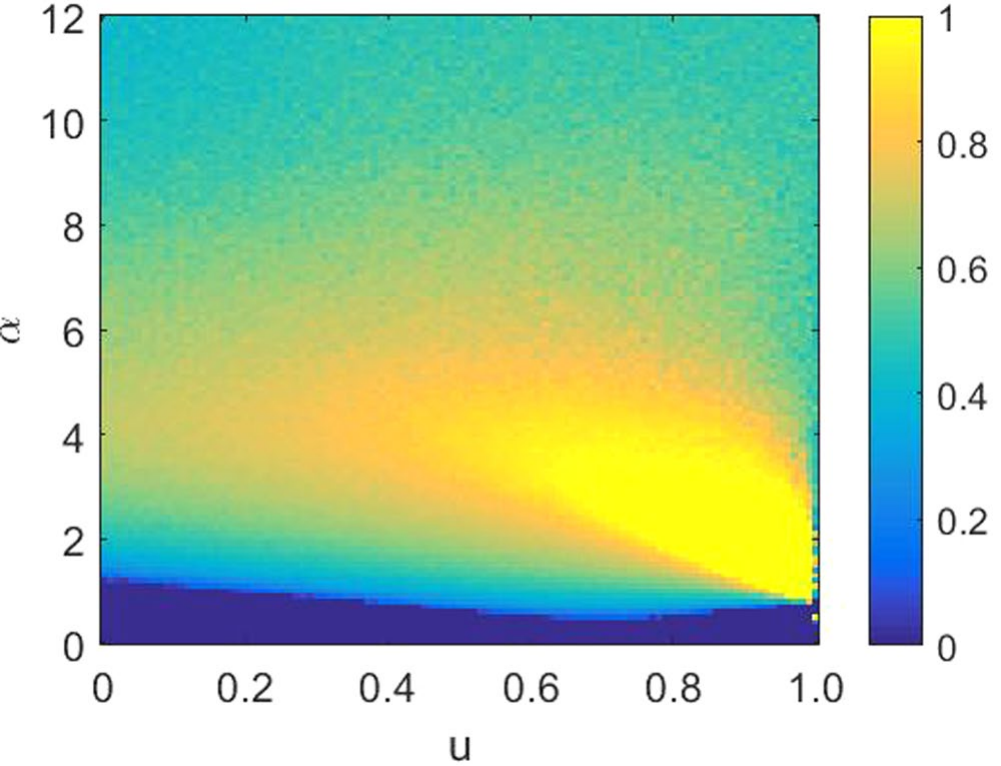
The color-coded (see bar on the right) fraction of cooperation *ρ*_*c*_ on the *α*-*u* parameter plane. When *α* = 0 and *u* = 0, it will turn to the traditional game, where cooperators go extinct soon. However, when the heterogrneity preference is considered, the fraction of cooperation can be promoted obviously with the increase of parameter *α* and *u*. In particular, for each fixed *u* value, the optimal *α* value for promoting cooperation lies between 2.0 and 6.0. All the results are obtained for *b* = 1.05, *L* × *L* = 10^4^ nodes, *k* = 4 and *K* = 0.1.

In the above section we have investigated how cooperation evolves when either the parameter *α* or the parameter *u* is fixed. In order to more fully demonstrate the impact of the combination mechanism on the evolution of cooperation, we present in Fig. 5 the color map encoding the fraction of cooperation *ρ*_*c*_ on the *α*-*u* parameter plane. The temptation to defect *b* is set at 1.05. When *α* = 0 and *u* = 0, the model will turn to the traditional game, where each neighbor of the focal individual is selected at random for strategy imitation and all the agents are homogenous. For the chosen value of *b*, cooperators die out as expressed in the blue area. In fact, cooperation exists across the entire parameter plane except for a very small parameter space. More intuitively, almost the entire plane is filled with yellow or light yellow color representing a high density of cooperation, as displayed in the graph, which confirms the conclusion that the combination mechanism is more conducive to the evolution of cooperation. The best parameter configuration to promote cooperation, as indicated by the yellow area, is consistent with the above.

**Figure 6:**
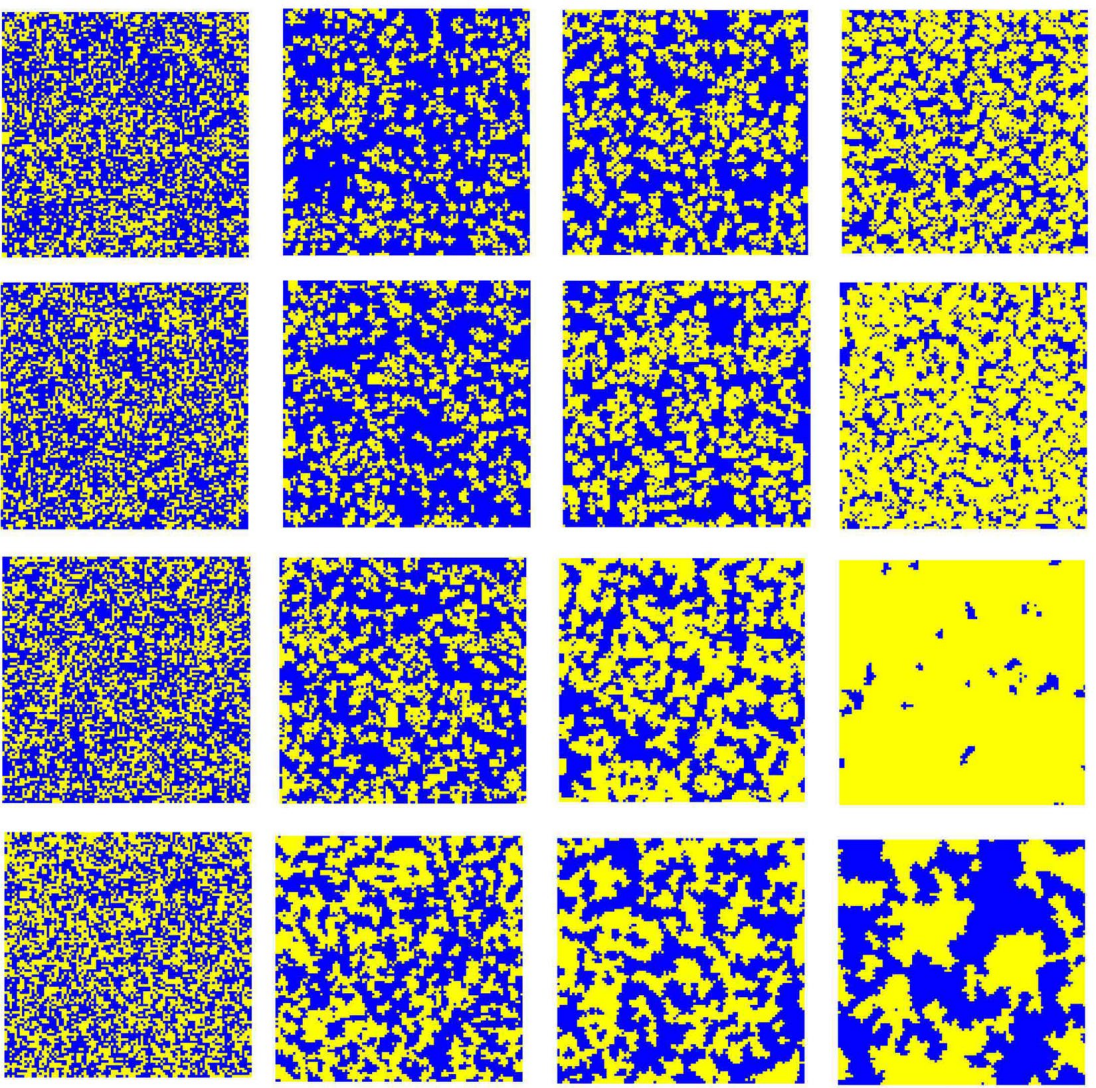
Snapshots of the distribution of cooperators (yellow) and defectors (blue) for *α* = 3.0. From left to right, the MCS steps are 0, 10, 100 and 10000, respectively. From top to bottom, *u* are equal to 0, 0.4, 0.8, and 1.0, respectively. The temptation to defect *b* = 1.1 and other parameters are the same as those in Fig. 5.

It remains of great interest to explore the spatial distribution of strategies under the introduced mechanism for different MCS steps and the results are presented in Fig. 6. It is instructive to give an understanding of the reason that this mechanism favors the evolution of cooperation. The cooperators and defectors are marked by yellow and blue, respectively. From top to bottom, the values of *u* are equal to 0, 0.4, 0.8 and 1.0, respectively. From left to right, the MCS steps are 0, 10, 100 and 10000, respectively. It should be pointed out that when the MCS is 10000, the system is in the evolutionary stable state. The temptation to defect *b* is fixed at 1.1 and *α* is set to be 3.0. At the beginning of evolution, cooperators and defectors are randomly distributed on the square lattice, as demonstrated in the first column. When *u* = 0.0, although the heterogeneous environment does not contribute to the fitness of the agent, there are still some cooperators in the evolutionary stable state under the preference selection mechanism, as indicated in the last column of the first row. As the value of *u* increases, more cooperators can survive by forming small clusters or patches which protect themselves from being exploited by defectors, as shown in the second row (*u* = 0.4). Compared with the case *u* = 0.0, we can observe that the clusters formed by cooperators become more compact and the distance among them is much smaller than the size of the clusters, which inevitably leads to less space for defectors. When the value of *u* continues to be increased, the formation and the spread of compact cooperation clusters become more and more obvious, which suppresses the invasion of defection. For example, when *u* = 0.8, few sporadic defector clusters survive and the cooperators dominate the population. It is interesting that when *u* = 1.0, as predicted before, the frequency of cooperators has been fluctuating around 0.5. However, in the processes of evolution, cooperators and defectors in equilibrium are not randomly distributed as they were in the initial stage. From the last row, we can clearly observe that the cooperators are constantly gathering separately, and finally some larger clusters are formed, although the proportion of cooperators has not changed significantly. It is easy to understand that when *u* = 1.0, although the evolution of individual strategies does not depend on the game gains, the introduction of heterogeneity of the environment and spatial structure enable cooperators to form clusters.

## Discussion

In conclusion, motivated by the realistic situation that the individuals are heterogeneous and the individual’s reference object selection is also different from person to person, we have investigated the impact of the combination of preference selection and environment heterogeneity on the evolution of cooperation in the spatial PDG. The simulation results show that either the preference selection effect or the environmental heterogeneity can enhance the evolution of cooperation to a certain extent. However, the promotion effect of cooperation is more pronounced under the mixed mechanism. In particular, when the parameter *u* is fixed at 0.5, we find that there exists an appropriate *α* value leading to the maximum value of cooperation for smaller *b* values. For larger *b* values, the number of cooperators increases first and then fluctuates around a certain value as *α* increases. Moreover, across the entire range of dilemma strength *b*, the promotion effect on cooperation is most obvious when the selection parameter *α* is between 2.0 to 6.0. Meanwhile, for the fixed parameter *α* (*α* = 3.0), the fraction of cooperation is positively related to the value of *u*. However, when *u* = 1.0, the fraction of cooperators fluctuates around 0.5 independent of the game process, which is determined by the composition of the individual’s fitness. To explore the mechanism that drives the evolution of cooperation, the snapshots of the distribution of cooperators and defectors with typical parameter configuration for different MCS steps are presented. The simulations suggest that the combination of the preference selection and the heterogeneity of the environment can be beneficial for accelerating microscopic organization of cooperative clusters, which becomes impervious to defector attacks even for a large value of *b*.

The above results can help us construct a comprehensive understanding of the effects of preference selection and heterogeneity on the evolution of cooperation under a simple framework of the spatial PDG. Actually, there are a plethora of suitable extensions that enable cooperative behaviors to persist. The investigations of the integration mechanisms may inspire more activities in this line of research for better cooperation promotion. Moreover, the environment in which an individual lives is evolving. Testing our model on coevolution scenarios, serving as the catalyst for the evolution of cooperation, will become more attractive in the future. Since both the phenomenon of preference choice and environmental heterogeneity are ubiquitous in reality, we hope that this work might provide additional insights for understanding the roots of cooperation and can inspire more exploration for resolving the social dilemmas.

## Methods

We consider an evolutionary prisoner’s dilemma game, in which the players populate on the vertices of the square lattice with periodic boundary conditions. As a standard practice, the evolutionary PDG is characterized with the temptation to defect *T* = *b* (the highest payoff obtained by a defector if playing against a cooperator), the reward for mutual cooperation *R* = 1, the punishment for mutual defection *P* = 0, and the sucker’s payoff *S* = 0 (the lowest payoff received by a cooperator when playing with a defector). It is worth mentioning that even if we choose a weak and simple PDG (namely, *P* = *S* = 0), our conclusions are robust and can be concluded in the full parameterized space.

The game is iterated forward in accordance with the Monte Carlo simulation procedure comprising the following steps. Throughout this work, each player *x* is designed either as a cooperator *s*_*x*_ = C or a defector *s*_*x*_ = D with equal probability. The size of the square lattice is 100 × 100. At each time step, a randomly selected player *x* first obtains its payoff *P*_*x*_ by playing the game with its nearest four neighbors. In this model, we assume that individuals are heterogeneous. Heterogeneity of individual *x* is expressed in *h*_*x*_ which is represented by a random number that is uniformly distributed on [0,4] with an interval of 0.1. Heterogeneous individuals lead to the diversity of individual neighborhoods, resulting in heterogeneous environments. Moreover, social interaction may not be the only way of generating payoffs. Therefore, we assume that the fitness of the player is affected by the environment. The environment that individuals live in is often determined by the average level of the characteristics of their neighbors. The heterogeneous environment *H*_*x*_ of individual *x* is thus defined as:

1 $${H}_{x}=\frac{{\sum }_{y=1}^{{k}_{z}}{h}_{x}}{{k}_{x}},$$

where the sum runs over all the neighbors of player *x*, and *k*_*x*_ represents the degree of player *x*. Then, we can calculate the fitness of player *x* in the following expression:

2 $${F}_{x}=(1-u){P}_{x}+u{H}_{x},$$

we suppose that the range of the parameter *u* goes from 0 to 1. Obviously, when *u* = 0, the model will turn to the traditional version. While when *u* ≠ 0, the heterogeneity of the individual is introduced. When the focal player *x* updates its strategy, it will select one neighbor *y*, who also acquires its fitness *F*_*y*_ in the same way, according to the following probability:

3 $${{\rm{\Omega }}}_{y}=\frac{\exp (\alpha {h}_{x})}{\sum _{z}\,\exp (\alpha {h}_{x})},$$

where *α* is the so-called preference selection parameter, and the sum runs over all the neighbors of player *x*. Evidently, from Eq. (3), *α* = 0 returns to the frequently adopted case where the neighbor *y* is randomly chosen. Lastly, player *x* tries to adopt the strategy of the selected neighbor *y* with a probability depending on the fitness difference,

4 $${W}_{y\to x}=\frac{1}{1+\exp ([({F}_{x}-{F}_{y})]/K)},$$

where *K* denotes the amplitude of noise or its inverse (1/*K*), the so-called intensity of selection^70^. Since the impact of *K* has been extensively investigated, we set the value of *K* to 0.1 in this work. During one full iteration step each player has a chance to adopt one of its neighbors’ strategies once on average.

The key quantity the fraction of cooperators *ρ*_*c*_ is determined by averaging the last 10^4^ full MCS (Monte Carlo simulation) over the total 6 × 10^4^ steps. To assure that the system has reached a stationary state, we analyze the size of the fluctuations in <*ρ*_*c*_>. If the size is smaller than 10^−2^, we suppose that the stationary state has been reached. Otherwise, we wait for another 10^4^ time-steps and redo the check. Actually, the system reaches the stationary state in all the simulations and no extra time-steps are needed. Moreover, since the heterogeneous environment may introduce additional disturbances, all the results are averaged over 40 independent runs for each set of parameter values in order to assure suitable accuracy.
